# Multidisciplinary Delphi Panel on Rehabilitation Approaches and Unmet Needs for Chronic Stroke Walking Impairment and the Role of Rhythmic Auditory Stimulation

**DOI:** 10.7759/cureus.68336

**Published:** 2024-08-31

**Authors:** Dorian K Rose, Carolee J Winstein, Michael D Lewek, Prudence Plummer, David J Lin, Holly Roberts, Preeti Raghavan, Sabrina R Taylor, Kirsten E Smayda, Michael W O'Dell

**Affiliations:** 1 Department of Physical Therapy, Health Science Center, University of Florida, Gainesville, USA; 2 Brain Rehabilitation Research Center, Malcom Randall Veterans Affairs Medical Center, Gainesville, USA; 3 Research Department, Brooks Rehabilitation, Jacksonville, USA; 4 Division of Biokinesiology and Physical Therapy, Herman Ostrow School of Dentistry, University of Southern California, Los Angeles, USA; 5 Department of Neurology, Keck School of Medicine, University of Southern California, Los Angeles, USA; 6 Division of Physical Therapy, Department of Health Sciences, University of North Carolina at Chapel Hill, Chapel Hill, USA; 7 Department of Physical Therapy, MGH Institute of Health Professions, Boston, USA; 8 Department of Neurology, Division of Neurocritical Care and Stroke Service, Center for Neurotechnology and Neurorecovery, Massachusetts General Hospital, Boston, USA; 9 Department of Neurology, Harvard Medical School, Boston, USA; 10 Medical Affairs, Independent Contractor, Philadelphia, USA; 11 Department of Physical Medicine and Rehabilitation and Department of Neurology, Johns Hopkins University School of Medicine, Baltimore, USA; 12 Department of Medical Affairs, MedRhythms, Inc., Portland, USA; 13 Independent Consultant, NeuroRehabilitation Consultants, New York City, USA; 14 Department of Rehabilitation Medicine, Weill Cornell Medicine, New York City, USA

**Keywords:** auditory cueing, walking, rhythmic auditory stimulation, outpatient rehabilitation, motor recovery, modified delphi, intervention, gait impairment, chronic stroke

## Abstract

Introduction: Walking or gait impairment is a common consequence of stroke that persists into the chronic phase of recovery for many stroke survivors. The goals of this work were to obtain consensus from a multidisciplinary panel on current practice patterns and treatment options for walking impairment after stroke, to better understand the unmet needs for rehabilitation in the chronic phase of recovery and to explore opportunities to address them, and to discuss the potential role of rhythmic auditory stimulation (RAS) in gait rehabilitation.

Methods: A panel of eight experts specializing in neurology, physical therapy, and physiatry participated in this three-part, modified Delphi study. Survey 1 focused on gathering information to develop statements that were discussed and polled during Survey 2 (interactive session), after which revised and new statements were polled in Survey 3. Consensus was defined as ≥75% (6/8 of panelists) agreement or disagreement with a statement.

Results: Consensus agreement was ultimately reached on all 24 statements created and polled during this process. The panelists agreed that individuals with gait or walking impairment in the chronic phase of stroke recovery can achieve meaningful improvement in walking by utilizing various evidence-based interventions. Barriers to treatment included cost, access, participation in long-term treatment, and safety. Consensus was achieved for interventions that have the following features challenging, personalized, accessible, and engaging. Improvement of gait speed and quality, durability of effect, safety, affordability, and ability for home or community use also emerged as important treatment features. In addition to conventional treatments (e.g., physical therapy, including mobility-task training and walking/exercise therapy), RAS was recognized as a potentially valuable treatment modality.

Discussion: This panel highlighted limitations of current treatments and opportunities to improve access, participation, and outcomes through a consideration of newer treatment strategies and patient/healthcare provider education and engagement.

## Introduction

Gait abnormalities and walking impairment (e.g., decreased speed and reduced walking capacity) are common consequences of stroke [1,2]. The persistence of walking impairment in the chronic phase of stroke recovery has been shown to decrease independence in activities of daily living, impair quality of life, and increase risk of morbidity and mortality [3-6]. Evidence suggests that resolution of walking impairment in the subacute phase of recovery is limited without treatment. One retrospective medical record review revealed that, upon discharge after approximately 25 days of inpatient rehabilitation, fewer than one-third of stroke survivors met criteria for independent community ambulation [7]. As many as 25% of stroke survivors are unable to walk independently in the chronic stage of recovery [8,9], and >50% experience continued gait limitations [10,11]. Furthermore, a substantial proportion of individuals with chronic stroke undergo a significant deterioration in mobility over time [12]. The limited degree of spontaneous improvement (i.e., without intervention) in chronic stroke and the negative consequences of walking impairment and reduced mobility among stroke survivors are well documented [3-6]. 

Common interventions for chronic stroke walking, or gait impairment include various forms of therapeutic exercise [13], with national and international treatment recommendations and clinical practice guidelines generally agreeing that moderate- to high-intensity walking training should be included in rehabilitation programs for stroke survivors [14-17]. Guidelines are less consistent for interventions such as virtual reality (VR)-coupled treadmill or balance training, rhythmic auditory stimulation (RAS), transcranial electrical stimulation, or robot-assisted therapy, as exemplified by differences in strength or nature of recommendations for these treatments based on awareness or interpretation of available evidence [14-17]. Persistent walking impairment in the acute/subacute phase for people recovering from stroke results in a substantial burden on survivors and their caregivers and healthcare systems [7]. Therefore, a key unmet need for chronic stroke survivors is for interventions that improve long-term mobility and ambulation status [1,12,18,19]. Increased awareness and implementation of a broad range of interventions may help to improve the outcomes of stroke rehabilitation.

The panel was funded by MedRhythms, Inc., a company developing products utilizing the principles of RAS to improve walking in people with neurologic disease or injury. RAS is an intervention based on principles of motor learning that can facilitate rehabilitation for individuals with chronic stroke walking or gait impairment, through involuntary synchronization of the timing of stepping movements with the timing of a rhythmic auditory stimulus [20-22]. RAS is extrinsically driven (using an auditory cue or rhythmic stimulus) toward accomplishing a goal (e.g., maintaining a rhythm while walking) using a particular protocol to increase or decrease the tempo of music while maintaining gait quality [20]. As such, RAS represents a different treatment modality emergent from a fundamentally different scientific basis (i.e., extrinsically driven auditory motor entrainment).

Here, we report on the outcomes of a multidisciplinary Delphi panel of clinical experts in chronic stroke rehabilitation. The directives of the panel were to 1) assess, discuss, and obtain consensus on current practice patterns and treatment options for walking or gait impairment in individuals with chronic stroke; 2) better understand the unmet needs for continued rehabilitation in that setting; 3) explore opportunities to address current limitations of gait recovery during the chronic phase of stroke recovery; and 4) discuss RAS, a potentially underutilized treatment option, as a treatment for chronic stroke walking or gait impairment.

## Materials and methods

Panel overview

This was a three-part, modified Delphi panel process conducted with participation of clinical experts currently working in various aspects of stroke treatment and rehabilitation; specialties included neurology, physical therapy, and physiatry. Based on clinical expertise and experience, potential participants were selected from a list generated in consultation between the study sponsor (MedRhythms, Inc., Portland, ME) and study administrator (Eversana, Burlington, ON), with the goal of creating a panel covering critical and diverse clinical roles (in public and private practices) across stroke treatment, clinical research, and rehabilitation. No ethics approval or consent was required for this Delphi panel study.

The Delphi technique involves asking a panel of experts their opinion on an issue, summarizing and presenting their responses, and repeating the process for multiple rounds [23]. Key features of a modified Delphi panel include iteration (allowing for rounds of feedback for participants), controlled feedback (allowing vocalization of collective opinions and judgements), and statistical aggregation (allowing for the presentation of the summary of the group response) [23]. In accordance with most Delphi panels in health sciences, which generally include eight to 20 experts to ensure meaningful discussions, achievement of consensus, and manageability of data collected [23], invitations were distributed by the study administrator with a target panel size of eight to 12 participants. Based on responses (agree, decline, and no response), a total of eight experts were engaged for this Delphi panel, with two panelists (DKR and MWO) also serving as a panel steering committee that reviewed and provided feedback on the surveys before each round was conducted.

Panel surveys and consensus statements

**Figure 1:**
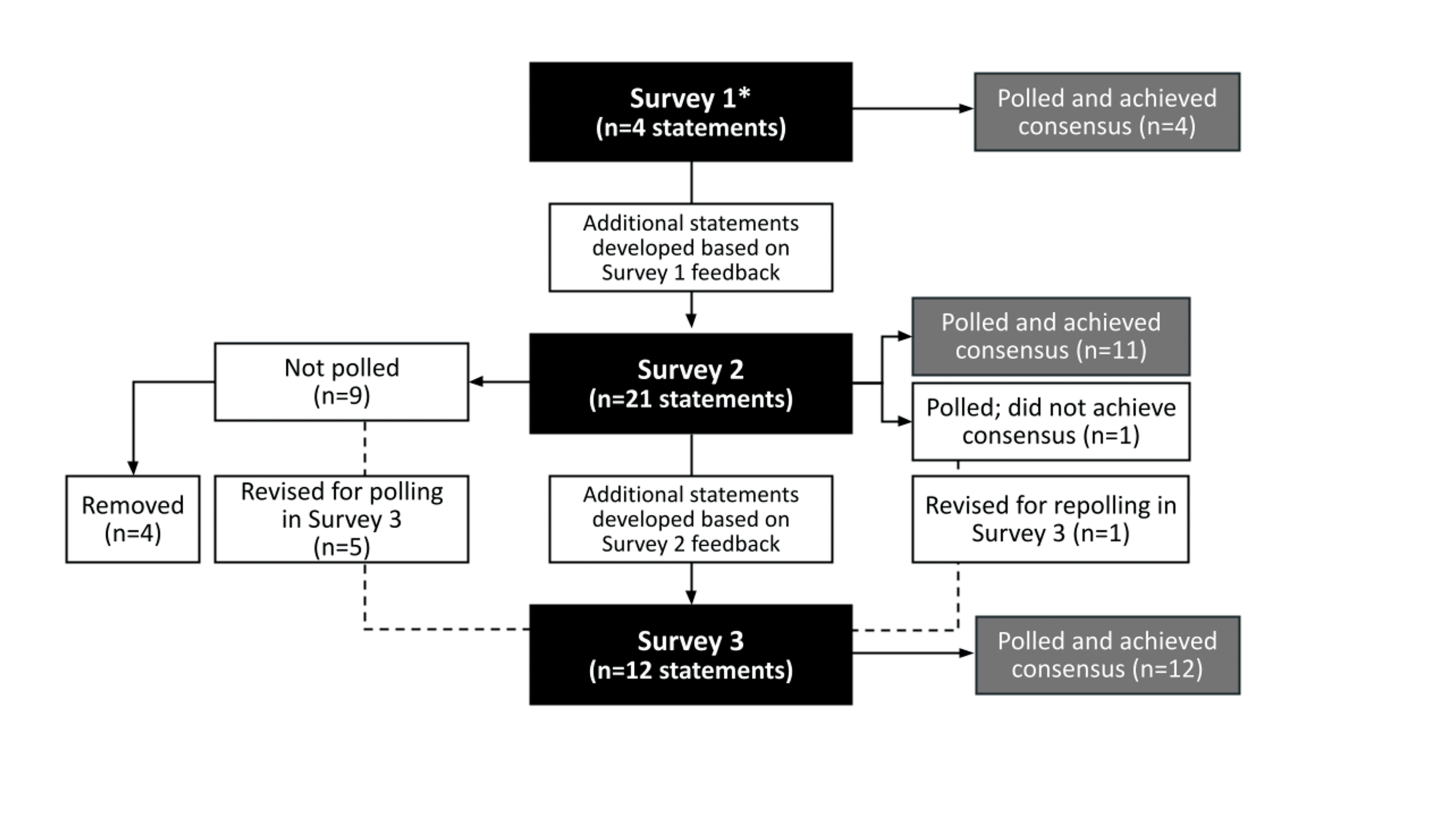
Delphi process flow chart Panelist feedback was applied throughout the process to inform development, revision, or removal of statements. Statements reaching consensus agreement were not re-polled in subsequent rounds. *Survey 1 included four statements and 11 open-ended questions and two rating-scale questions for information-gathering purposes.

The study consisted of three rounds of survey-based polling completed over five months. A flow chart of the development and progress for consensus statement building throughout the Delphi process is illustrated in Figure 1. Surveys 1 and 3 were administered in an electronic format and developed and distributed using the Forsta HX Survey platform (Forsta AS, Oslo, Norway). Survey 2 was administered in a virtual live panel discussion session with all eight panelists in attendance. For the purposes of all panel surveys and discussions, walking or gait impairment was defined as any deviation from “normal” walking, including decreased speed, decreased and asymmetrical step length, decreased stance phase, and/or altered joint kinematics. When responding to the surveys, the clinical experts were instructed to focus on their experiences and thoughts related to the treatment of ambulatory individuals after stroke in the chronic stage of recovery. For all statements, the level of agreement was rated as “strongly disagree,” “disagree,” “neutral,” “agree,” or “strongly agree”; consensus was defined as ≥75% agreement or disagreement. Statements reaching consensus in a given survey were not revisited in subsequent surveys unless otherwise noted. 

Survey 1 was developed by the study sponsor, study administrator, and steering committee based on a comprehensive assessment of published literature with the primary purpose of gathering data from the panel, which was subsequently used to generate statements for consensus polling. The goal of this survey was to gain an understanding of how experts in different specialties who treat or conduct research with individuals with chronic stroke perceive current treatment options for walking or gait impairment. Survey 1 (completed in May 2023) consisted of 11 open-ended questions related to clinical experience and perceptions; two rating-scale questions focused on clinical benefit and importance of various interventions and treatment features (rated as “low,” “minimal,” “neutral,” “moderate,” “high,” or “not applicable”); and one rating-scale question evaluated the level of agreement with four statements developed based on unmet needs in treatment of chronic stroke walking impairment identified from published literature. Individual questions and statements captured in Survey 1 are detailed in Appendix A.

Survey 2 (completed during a three-hour virtual live panel discussion in June 2023) consisted of 21 statements developed based on the responses to Survey 1. For example, panelist responses to question 4a in Survey 1 “… describe your current treatment practices related to walking and/or gait impairment” informed statement #6 in Survey 2, “the most common treatments for walking/gait impairment in individuals with chronic stroke are….” The goal of Survey 2 was to achieve consensus or, where consensus could not be reached, revise statements in real time and re-poll during the live session. Some polled and unpolled statements were deferred for offline revision (e.g., based on the complexity of feedback/revisions or evolution/flow of the discussion) and were revisited in Survey 3. Individual statements discussed in Survey 2 are detailed in Appendix B.

Survey 3 (completed electronically in September 2023) consisted of 12 statements generated from Survey 2 feedback with the goal of reaching final consensus. These included revised statements that were either not polled, or did not reach consensus during Survey 2, and new statements developed based on conversations during the virtual live panel discussion. New statements generated per the standard Delphi process were related to the importance of considering gait endurance and improvement in daily walking in treatment. In addition, the sponsor was interested in the awareness of RAS as a viable therapeutic intervention among clinicians who treat individuals with chronic stroke walking or gait impairment. Individual statements polled in Survey 3 are detailed in the Appendix. Unlike Survey 1 and Survey 2, a scientific and clinical assessment brief concerning RAS was provided to the panel prior to initiating Survey 3 to ensure that all participants had the same information about the clinical intervention (see Appendix C). The brief included information on the definition, theory, and biological principles of RAS, a RAS clinical protocol describing therapy “dosing” and procedures for stroke treatment [20], and a table of RAS clinical trials in individuals with stroke. RAS trials were selected for inclusion based on two peer-reviewed meta-analyses assessing RAS for impaired gait after stroke [21,24]. RAS trials conducted after 2019 in individuals post-stroke who had walking or gait impairment were also included for panelist review. In addition to the brief provided after Survey 2, highlights of key information were provided in the electronic survey before polling on RAS-focused statements. 

Data analysis

No formal statistical analyses were performed for the results of this Delphi panel, which were summarized descriptively. Responses to Survey 1 questions were compiled as text for open-ended questions and summarized numerically for rating-scale questions. For the ranking of the clinical benefit of various treatments in Survey 1, top answers were selected based on all panelists ranking the treatment as “high,” “moderate,” or “neutral” (excluding responses of “not applicable”). Similarly, for the ranking of the importance of various treatment features, top answers were selected based on all panelists ranking the treatment feature as highly or moderately important. For both rating-scale questions, written-in answers with only one responding panelist were not included to avoid over-interpretation. As noted above, for all statements polled, consensus was considered achieved if six or fewer of the eight panelists (or ≥75%) were aligned in their agreement or disagreement of a statement. Agreement was defined as an answer of “agree” or “strongly agree,” while disagreement was defined as an answer of “disagree” or “strongly disagree.”

## Results

The panelists were experts in stroke rehabilitation (research and clinical treatment) and credentialed as MDs (neurology, n = 2; physiatry, n = 2) or physical therapists (n = 4). The extent of experience (clinical practice and/or research) ranged from seven to 50 years, with a mean ± SD of 28.8 ± 13.4 years and representing 259 cumulative years across panelists. Of the eight panelists, seven fully completed Survey 1 and all eight completed Survey 2 and Survey 3. The panelist who partially completed Survey 1 responded only to polling questions #1-3; they were therefore excluded from data analysis from question #4 onward. The panelists reported treating individuals who had experienced a stroke ranging from within a few hours to 30 years post-stroke. Of the seven panelists who reported patient treatment settings, four reported treatment in both inpatient and outpatient settings (with one also including home/residential care/assisted living settings); two panelists reported only the outpatient setting, and one reported a research setting. All questions and statements polled during the three surveys are detailed in the tables in the Appendix, and all statements achieving consensus are presented in Table 1. Consensus statements with the highest strength of agreement, defined as >75% of the panelists “strongly agree” with the presented statement, were #9 (100%), #8 (87.5%), #5 (86%), #12 (75%), and #13 (75%).

**Table 1:** Final Consensus Statements RAS, rhythmic auditory stimulation. *Survey 1 was completed by 7/8 panel members; Surveys 2 and 3 were completed by all 8 panelists. †One vote was missing for question 5 in Survey 2.

	Statement number	Statement	Agreement, % (rank)	Consensus
Survey 1*	1	There is opportunity for improvement in the treatment of walking and/or gait impairments in chronic stroke	14 (strongly agree) 86 (agree)	Consensus achieved (agreement)
	2	There are limitations associated with the current treatment options that may result in suboptimal patient outcomes	29 (strongly agree) 71 (agree)	Consensus achieved (agreement)
	3	There are limitations associated with the current care that may result in inequities in patient access	29 (strongly agree) 71 (agree)	Consensus achieved (agreement)
	4	There is an unmet need for a treatment option that addresses some of the limitations associated with the current care	43 (strongly agree) 57 (agree)	Consensus achieved (agreement)
Survey 2	5^†^	For individuals with walking and/or gait impairments in chronic stroke, there is still potential for further recovery with continued, intentional intervention	86 (strongly agree) 14 (strongly disagree)	Consensus achieved (agreement)
	6	The most common treatments for walking/gait impairment in individuals with chronic stroke are overground gait training, treadmill training, balance training, and strength training, typically administered by a physical therapist	50 (strongly agree) 50 (agree)	Consensus achieved (agreement)
	7	There are critical barriers in treating walking/gait impairment in chronic stroke, including poor adherence, lack of accessibility (due to travel needs and availability of advanced treatment options), lack of insurance coverage, and concerns about safety	50 (strongly agree) 50 (agree)	Consensus achieved (agreement)
	8	There is a need for improved education, so that individuals understand treatment goals, expected treatment progress, and the potential value of treatment, which may help to optimize engagement and participation	87.5 (strongly agree) 12.5 (agree)	Consensus achieved (agreement)
	9	Participation in treatment could be improved if individuals clearly understand how the treatment helps, see benefits associated with treatment, and find the treatment enjoyable	100 (strongly agree)	Consensus achieved (agreement)
	10	The most critical features for a valuable treatment option for walking and/or gait impairment in chronic stroke include durability of treatment effect; safety; improvement in walking/gait speed, quality, endurance, and cosmesis; and treatment accessibility	50 (strongly agree) 37.5 (agree) 12.5 (strongly disagree)	Consensus achieved (agreement)
	11	A potentially valuable therapy for treating walking and/or gait impairments in chronic stroke would be personalized to the individual, while being progressively challenging	12.5 (strongly agree) 87.5 (agree)	Consensus achieved (agreement)
	12	A potentially valuable therapy for treating walking and/or gait impairment in chronic stroke would provide periodic feedback about progress and support personal goal setting, which may help foster continued engagement in the treatment	75 (strongly agree) 25 (agree)	Consensus achieved (agreement)
Survey 3	13	In the chronic phase of stroke, motor recovery is possible with appropriate interventions that challenge the individual at the appropriate level	75 (strongly agree) 25 (agree)	Consensus achieved (agreement)
	14	In the chronic phase of stroke, a plateau in motor recovery may be an artifact resulting from discontinued interventions and/or the measurement tools currently available to clinicians	37.5 (strongly agree) 50 (agree) 12.5 (disagree)	Consensus achieved (agreement)
	15	Effectively treating walking and/or gait impairments in individuals with chronic stroke should be a priority	37.5 (strongly agree) 62.5 (agree)	Consensus achieved (agreement)
	16	Key considerations when devising a treatment plan for walking and/or gait impairments for individuals with chronic stroke include intervention efficacy, ambulatory status, patient independence, patient preference, insurance coverage, and caregiver availability	37.5 (strongly agree) 62.5 (agree)	Consensus achieved (agreement)
	17	Treatment options that may provide moderate/high clinical value for treating walking/gait impairments in chronic stroke, based on principles of motor relearning, are general/combination physical therapy, therapeutic exercise, mobility-task training, and rhythmic auditory stimulation (RAS)	25 (strongly agree) 50 (agree) 12.5 (neutral) 12.5 (disagree)	Consensus achieved (agreement)
	18	The US healthcare system structure imparts a critical barrier to following individuals with chronic stroke once discharged from formal acute rehabilitation, resulting from fragmented, uncoordinated care from multiple clinicians throughout the rehabilitation journey	62.5 (strongly agree) 37.5 (agree)	Consensus achieved (agreement)
	19	An unmet need exists for therapeutic interventions that can be practiced safely outside of the clinic to improve treatment accessibility and participation	62.5 (strongly agree) 25 (agree) 12.5 (neutral)	Consensus achieved (agreement)
	21	A valuable intervention for treating walking and/or gait impairments in chronic stroke would track gait quality metrics and walking speed and/or distance	37.5 (strongly agree) 50 (agree) 12.5 (disagree)	Consensus achieved (agreement)
	21	Individuals with walking and/or gait impairment in chronic stroke should have access to a treatment plan that includes in-clinic and at-home practice with the goal of gradually increasing at-home practice while decreasing in-clinic practice as the individual progresses	62.5 (strongly agree) 25 (agree) 12.5 (neutral)	Consensus achieved (agreement)
	22	The available evidence supports RAS as an effective therapy for improving walking and/or gait speed and quality (step cycle, step length, cadence, etc.) in individuals with chronic stroke	12.5 (strongly agree) 62.5 (agree) 25 (neutral)	Consensus achieved (agreement)
	23	A treatment mechanism to deliver individualized RAS in a device that can be used safely at home could be valuable for individuals	12.5 (strongly agree) 75 (agree) 12.5 (neutral)	Consensus achieved (agreement)
	24	There is an opportunity for education on and implementation of RAS amongst clinicians who treat individuals with chronic stroke	25 (strongly agree) 62.5 (agree) 12.5 (neutral)	Consensus achieved (agreement)

Survey 1

**Figure 2:**
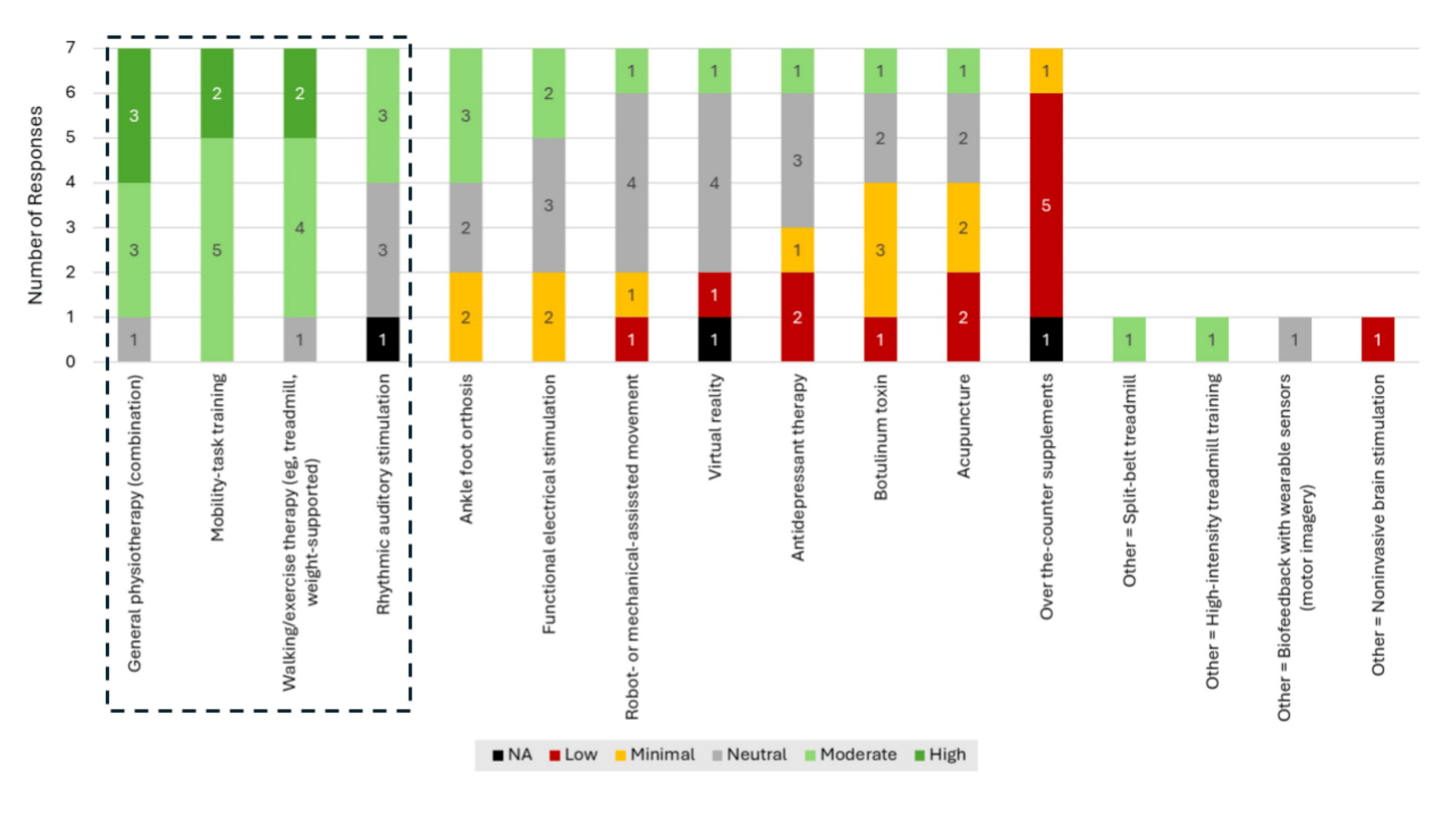
Panel Ranking of Clinical Benefit of Current Treatments In Survey 1, panelists rated the clinical benefit associated with 16 treatment options for individuals with walking or gait impairment in chronic stroke. Treatments representing the focus of open discussions during the live Survey 2 session are indicated with black dashed outline. NA, not applicable.

All eight panelists defined chronic stroke (question #2) as >six months after the stroke event, consistent with the framework proposed by a 2017 Stroke Recovery and Rehabilitation Roundtable [8]. Write-in remarks in the context of chronic stroke highlighted diminishing access to, or discontinuation of, rehabilitation treatment in the chronic phase and perceived recovery plateaus or slowing of further spontaneous recovery as important considerations for chronic stroke. The most common current treatment practices among panelists (question #3) were physical therapy and therapeutic exercise, including gait training overground and gait training on a treadmill, which are commonly performed by a physical therapist in an outpatient setting (though gait training can be performed in an inpatient setting). The panelists reported familiarity with many variations of gait training on a treadmill (including with and without body-weight support, with biofeedback, and walking on a split-belt treadmill) and reported several other current practices including balance, strength, endurance, and high-intensity interval training; nerve stimulation; botulinum toxin treatment; and pre-gait activities. Ratings of the potential clinical benefit for 16 existing treatments for walking impairment extracted through a comprehensive assessment of published literature (question # 5a) revealed the highest perceived benefit for general physiotherapy (high, n = 3/7; moderate, n = 3/7), mobility-task training (high, n = 2/7; moderate, n = 5/7), and walking/exercise therapy (high, n = 2/7; moderate, n = 4/7), followed by RAS and ankle foot orthosis (Figure 2). Regarding RAS specifically, the perceived benefit was overall positive, but the rankings were split between “moderate” and “neutral” (n = 3 each), with one panelist responding “not applicable.” Among newly available or emerging treatment options and their potential value (question #5b), the panelists reported telerehab, medications that enhance motor learning/memory, spinal cord and vagus nerve stimulation, and brain-computer interfaces for those who are severely impaired.

**Figure 3:**
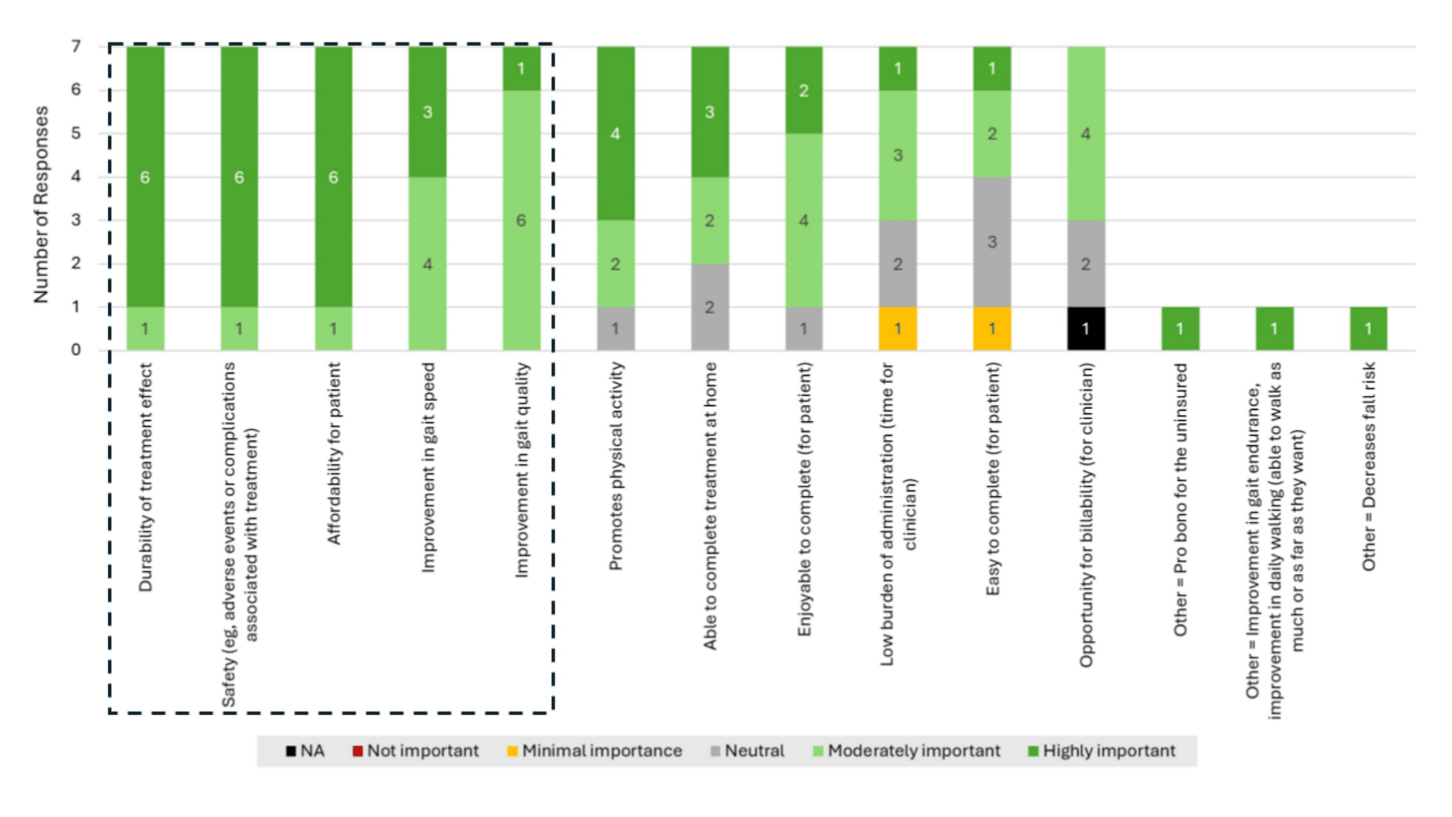
Panel Ranking of Features of a Valuable Treatment In Survey 1, panelists rated the importance of various features of treatment for individuals with chronic stroke walking or gait impairment. Treatment features identified as most important in the open discussions during the live Survey 2 section are indicated with black dashed outline. NA, not applicable.

Based on their experience, the panelists estimated that patient participation in their treatments as prescribed ranges from 50% to 100%, without a clear pattern across treatment strategies (question #4b). Most panelists (5/7) indicated that patient and clinician perspectives on treatment value (question 5c) may differ. For example, a patient may not perceive the value of a clinician’s recommendation as high if the treatment does not align with that individuals’ goals or is perceived to be too large a burden (e.g., too much time, effort, and/or cost) in the context of expected results. Key barriers and limitations to treatment (question #6a) included cost, transportation, provider access, and difficulty of treatment. The most highly rated important features of a therapy (question #7) were largely focused on efficacy-based characteristics (e.g., durability of treatment effect (highly important, n = 6/7; moderately important, n = 1/7), improvement in gait speed (highly important, n = 3/7; moderately important, n = 4/7), and movement quality (highly important, n = 1/7; moderately important, n = 6/7)). Additional features included safety of the treatment (highly important, n = 6/7; moderately important, n = 1/7) and characteristics that may overcome potential barriers to treatment (e.g., affordability (highly important, n = 6/7; moderately important, n = 1/7)) (Figure 3). Write-in remarks (question #8) also highlighted the need for treatments to be available for home and community use and to be individualized and progressively challenging.

Question #9 asked the panelists to rate their level of agreement on four statements focused on unmet needs related to treatment limitations and opportunities to improve treatment and outcomes (Table 1). Consensus was achieved for all four statements, with 100% of panelists (n = 7/7) agreeing or strongly agreeing with these statements. 

Survey 2 and virtual live discussion

Of the 21 statements generated for Survey 2 based on feedback from Survey 1, 11 achieved consensus. Among the remaining 10 statements, one that was polled did not achieve consensus; based on the flow and focus of open discussions and panelist feedback, five statements were not polled and were revised offline for Survey 3, and four statements were removed altogether (Figure 1). The statement that did not reach consensus (#10 in Appendix B; “An unmet need exists for treatments that can be delivered at home by a non-clinician to improve treatment accessibility and adherence, ensuring the regular practice required to obtain meaningful change in walking speed”) received diverse ratings (12.5% strongly agree, 50% agree, 12.5% neutral, and 25% disagree). Feedback highlighted differences in patient goals for walking (e.g., based on acceptable threshold before shift in goal to increased duration or based on need/function such as keeping up with a grandchild or crossing the street) and the importance of access to treatment in the home setting while recognizing the value of in-clinic treatment. This statement was revised based on the panelist feedback for re-polling in Survey 3 (#7 in Appendix C). Two statements (#15 and #16 in Appendix B) reached consensus agreement but were reworded for re-polling as new statements in Survey 3 (#5 and #8 in Appendix C and #17 and #21 in Table 1).

Of the statements removed during Survey 2 (Appendix B), one relating to population heterogeneity and lack of availability of direct comparative evidence (#5) was removed because of updated clinical practice guidelines that addressed the concerns of this statement [14]. Statement #17, regarding treatments that have unclear/low clinical benefit, was removed because of lack of therapeutic context (for VR) and lack of practitioner familiarity (with functional electrical stimulation). Statement #18, regarding the clinical benefit of robot- or mechanical-assisted devices, was removed owing to technological advances addressing barriers of these treatments (e.g., bulkiness and cost). Lastly, statement #19, regarding the clinical benefit of ankle foot orthosis, was removed owing to the availability of choices that address the concerns regarding cosmesis and limited shoe options.

Similar to the Survey 1 feedback, the discussions and Survey 2 feedback in the live-virtual session emphasized a focus on physical therapy, including gait training overground and gait training on a treadmill. Additional considerations included the use of combination treatment strategies versus single-modality, standalone treatment. In the context of the clinical benefit of various treatment approaches ranked in Survey 1, the panelists indicated that strategies considered to provide high or moderate clinical value “should be based on the principles of motor learning” and/or exercise physiology. In the virtual live discussion, the panelists discussed that RAS-based therapy is a treatment that could help achieve these treatment principles and goals in ambulatory stroke survivors. Despite the overall positive benefits of RAS, perceptions and considerations related to RAS, including its definition, mode of delivery, protocol, and interpretation of any outcomes, highlighted an opportunity for clarification before revisiting the topic in Survey 3. In further discussion related to specific treatments, the panelists commented that VR is a vehicle used to deliver therapy, which creates a challenge for evaluation in terms of outcomes but that VR with biofeedback can have clinical benefit. Ultimately, when discussing the value of current treatment options for individuals recovering from stroke, panelists emphasized that value is dependent on how the treatment is being used, which protocol is being followed, and the patient’s individual response. During this live discussion session, panelists noted that common treatments are typically administered by a physical therapist but that not all individuals have access to physical therapy services. Regarding engagement in rehabilitation, patient education and early and ongoing engagement were identified as useful strategies to understand individuals’ goals and encourage participation in their treatment.

Survey 3

Consensus (≥75%) agreement was achieved for all 12 statements polled in Survey 3 (Table 1); these included the six revised statements from Survey 2 and six new statements developed based on panel feedback during the virtual live discussion (Figure 1). Three of the new statements developed for Survey 3 (#10, #11, and #12 in Appendix C) focused specifically on RAS.

## Discussion

This multidisciplinary Delphi panel captured complementary perspectives from highly experienced experts in healthcare specialties who all have an interest in improving walking or gait impairment for individuals recovering from stroke. Based on the consensus statements with the highest strength of agreement (see consensus statements #9, #8, #5, #12, and #13 in Appendix A), key insights from the panel feedback were cross-specialty agreement that individuals with gait, or walking, impairment in the chronic phase of stroke recovery can achieve meaningful improvement with several evidence-based interventions. Specifically, these interventions should be sufficiently challenging, personalized to individual needs and evolving to meet individual performance (i.e., progressively challenging), and accessible for independent practice. Furthermore, the stroke survivor needs to be engaged and see value in participating in their treatment. Because of the considerable socioeconomic burden, impact on daily living, impaired quality of life, and increased risk of morbidity and mortality associated with motor impairment in chronic stroke, effectively treating chronic stroke walking or gait impairment should be prioritized [1,3-7,12,18,19,25]. The panelists agreed that spontaneous recovery in the chronic phase of recovery is rare, but they also clarified that this observation is specific to the absence of therapy and proposed that a hypothetical plateau in motor recovery in chronic stroke may represent a lack of appropriate rehabilitation treatment rather than a true plateau. In this context, one panelist stated that “…there is potential for further recovery and/or modification of movement patterns with the right intensity, duration, and type of intervention.” In addition, potential ceiling effects of measurement tools currently available to clinicians for assessment of progress were also identified as a contributing factor to potentially artificial “plateaus,” consistent with previous literature describing the need for standardized guidelines and tools for evaluating chronic stroke walking impairment and recovery [25,26]. Suboptimal sensitivity for relevant metrics, applicability within versus beyond the clinical setting, and lack of clarity regarding timing of assessment may contribute to limitations of these tools.

A current clinical practice guideline reported overground walking (91%), balance training (64%), and training on a treadmill (40%) as the most common treatment strategies used by physical therapists in the United States [14]. These trends were reflected in panelists’ perceptions of the clinical value of various treatments, with the greatest benefit attributed to combination physical therapy, walking/exercise therapy, and mobility-task training. Consistent with evidence-based US and Canada recommendations for rehabilitation in chronic stroke, the panelists rated the benefits of RAS-based treatments favorably [15,27]. RAS was considered, but not included, in the 2020 American Physical Therapy Association Clinical Practice Guideline for Stroke Management because the small number of randomized controlled trials (n = 3) was considered insufficient to inform a recommendation on its use [14]. With respect to key treatment features, those that were considered most important by the panelists included durability of effect, facilitation of individuals’ participation in their treatment, accessibility, safety, individualized and progressive nature of treatment, and ability to practice outside of the clinical setting. Treatments based on the principles of motor relearning and exercise physiology were also considered to provide moderate to high clinical value. One advisor stated that an optimal therapy for individuals with chronic stroke and walking, or gait impairment, would be “prolonged high-intensity interval training, paired with step activity monitors … to promote greater daily stepping.”

Notably, some of the most highly rated features were also represented in panelist-reported barriers to treatment, highlighting opportunities to improve treatment. As reflected in the consensus statements, efforts to educate and engage patients may overcome some participation barriers (e.g., patient perception of treatment success/value and lack of motivation and reinforcement). For example, patient-centered goal setting, particularly in the outpatient setting, is positively associated with stroke recovery during rehabilitation [28,29]. In the experience of the panelists, other barriers include treatment cost and access, transportation costs and effort, and concerns about safety during independent practice. These barriers are particularly apparent when considering that commonly used treatments are typically administered by a physical therapist but not all individuals have access to physical therapy services. One panelist stated that “current treatments are almost all restricted to a medical setting …. A safe, inexpensive treatment that could be applied by a non-clinician in a home setting would have an immediate, functionally significant impact.” The considerable overlap in important treatment features, barriers to treatment, and barriers to participation in the treatment is unsurprising and emphasizes the importance of addressing these challenges when considering the features of available or emerging treatments and developing individualized treatment plans to improve outcomes and diminish burden on patients, caregivers, and healthcare systems [1].

Some newer/emerging treatments (e.g., brain-computer interfaces, transcranial brain stimulation, and robot-assisted therapy) have shown promise for rehabilitation in chronic stroke but may be difficult to integrate into practice patterns owing to barriers stemming from cost or need for specialized training [30]. VR-based treatment delivery, particularly when biofeedback is incorporated, was viewed here and by others as clinically useful [1,14] and could help to overcome some barriers to access and participation in treatment, but it was noted that practitioner familiarity may be low. 

The panelists also discussed their understanding and utilization of RAS as a clinical intervention for chronic stroke walking or gait impairment. Both before and after the scientific and clinical assessment brief on RAS was provided to the panel, the panelists acknowledged the potential clinical value of RAS and noted that RAS may represent a useful tool to help achieve treatment principles and goals. This could include helping individuals to walk faster and achieve higher intensity training or aiding in strategic motor learning by encouraging an individual to step to a changing beat or rhythm. In addition, one panelist noted that the novelty value of RAS-based therapy may support use-dependent learning, thereby helping to achieve higher volumes of practice needed for therapeutic benefit and/or long-term adherence. Moreover, a treatment mechanism to deliver individualized RAS in a device that could be safely used at home was considered to provide potential value to individuals with chronic stroke walking or gait impairment. Nonetheless, as a panelist noted, the lack of healthcare provider awareness (particularly among physical therapists) of RAS as a treatment modality for rehabilitation in chronic stroke poses a barrier to its use. Specifically, limited awareness or understanding of supporting evidence and standard clinical protocols, combined with the small number of robust randomized controlled studies of RAS, and the lack of consistency regarding inclusion of RAS across treatment recommendations and practice guidelines [14,15,17,27], may contribute to underutilization. Panel feedback indicates that continued efforts to support understanding of RAS are warranted, as this may provide an opportunity to integrate RAS as an additional treatment modality into individualized treatment plans and improve treatment and outcomes in chronic stroke walking or gait impairment.

Limitations

The small number of participants, all of whom are located in the US, may limit the overall generalizability of panel results to other clinical professionals and different geographic regions. The limited sample size poses a challenge for describing this dataset, and in mirroring convention for reporting panel results as percentages, the authors acknowledge the need for caution in doing so. Furthermore, the definition of walking or gait impairment used for this panel was not comprehensive (e.g., the scientific literature pertaining to abnormal muscle activation patterns was not included).

Due to the panel’s discourse about RAS during the virtual live Survey 2 and the study sponsor’s specific interest in understanding how the panelists view RAS, RAS-focused statements were developed for polling in Survey 3, and a scientific and clinical assessment brief describing RAS standard protocols and supporting evidence materials [20,21,24] were provided to panelists prior to Survey 3 to ensure that all panelists had the same foundational knowledge about this clinical intervention. Scientific and clinical assessment briefs were not provided for other discussed treatments. The intent was not to promote or position RAS (or any treatment modality) as more or less valuable than other treatments; outcomes and consensus statements related to RAS should be interpreted in this context.

## Conclusions

Overall, this Delphi panel captured the clinical experience and perspectives of a group of clinical research and treating specialists with a wide range of clinical specialties in neurology, physical therapy, and physiatry. Importantly, this study highlights unmet needs and potential opportunities to enhance gait recovery outcomes for individuals with chronic stroke, an often underserved population. This work resulted in 24 consensus statements focused on the current practices, unmet needs, and limitations of current treatment options, as well as on features of valuable therapies and opportunities to improve treatment of individuals in the chronic phase of stroke recovery and experiencing walking or gait impairment. The panelists agreed that effectively treating walking impairment in chronic stroke is possible but requires rehabilitation strategies that are personalized, sufficiently challenging and progressive, benefiting to individuals’ participation in treatment, and accessible. RAS-based treatment was also recognized as a potentially valuable treatment modality and merits consideration for inclusion in treatment plans. There is an opportunity for educational efforts focused on the potential role of RAS in chronic stroke to raise awareness of supporting evidence and appropriate clinical protocols for the rehabilitation of gait or walking impairment.

## Appendices

Appendix A

**Table 2:** Questions Administered During Survey 1 *Clinical benefit was rated as “low,” “minimal,” “neutral,” “moderate,” or “high.” †Treatment features were rated as “not important,” “minimal importance,” “neutral,” “moderately important,” or “highly important.” ‡Agreement with statements was rated as “strongly disagree,” “disagree,” “neutral,” “agree,” or “strongly agree.”

Question number	Survey 1 questions (polled statements are in bold)
Key opinion leader experience	
1	Describe your clinical experience/current position
Definition of chronic stroke	
2	What does the term chronic stroke mean to you?
Patient characteristics	
3	Describe the average demographic characteristics associated with patients with walking and/or gait impairment post-stroke
Current treatment practices related to walking and/or gait impairment	
4a	Based on the patient population you described, describe your current treatment practices related to walking and/or gait impairment
Patient adherence/compliance and barriers	
4b	Provide any insight you have into the adherence/compliance with prescribed treatments For patients that do not utilize treatments as prescribed, can you provide a qualitative overview of what barriers to adherence may be? Do you have a sense of if patients engage in additional treatment options that were not initially prescribed?
Clinical benefit of treatments*	
5a	Of the listed treatment options, how much clinical benefit do you think they offer to patients with walking and/or gait impairment in chronic stroke? (see Figure 2)
Other valuable treatment options	
5b	Are there other treatment options that are newly available or coming to market that you see as being valuable for patients with walking and/or gait impairments and chronic stroke not listed above? If so, please describe
Patient vs clinician perspective	
5c	When considering the benefits associated with different treatment options, does the perceived value differ if considering the patient vs clinician perspective? If so, how?
Treatment barriers/limitations	
6a	What do you see as the primary limitations or barriers associated with the current treatment options for treating walking and/or gait impairments in chronic stroke patients?
Treatment selection	
6b	Do these limitations impact your selection of treatment for a patient with chronic stroke? If yes, how so?
Treatment inequities	
6c	Do these limitations or barriers (eg, geography, socioeconomic status) result in inequities to treatment access? If yes, how so?
Importance of treatment features^†^	
7	Please rate the importance of each feature of treatments for walking and/or impaired gait in chronic stroke (see Figure 3)
Valuable therapy in an ideal world	
8	In an ideal world, without limitations and barriers to access, what would you describe as a valuable therapy to support walking and/or gait impairment in chronic stroke, explain why, and explain how it differs from current care (eg, more sessions, equipment/devices to use at home)?
Unmet need^‡^	
9	Please rate your level of agreement with the following statements (all statements were polled). 1) “There is opportunity for improvement in the treatment of walking and/or gait impairments in chronic stroke.” 2) “There are limitations associated with the current treatment options that may result in suboptimal patient outcomes." 3) “There are limitations associated with the current care may that result in inequities in patient access." 4) “There is an unmet need for a treatment option that addresses some of the limitations associated with the current care.”

Appendix B

**Table 3:** Survey 2 Statements RAS, rhythmic auditory stimulation. *Agreement with statements was rated as “strongly disagree,” “disagree,” “neutral,” “agree,” or “strongly agree.”

Statement number	Survey 2 statements (polled statements in bold)*
Patient definition	
1	Walking and/or gait impairments are a common problem in individuals with chronic stroke, and efforts should be made to treat them as effectively as possible.
2	Chronic stroke (≥6 months post-stroke) has historically been associated with a plateau in functional improvement and persistent deficits, typically correlated with the completion of formal rehabilitation.
3	For individuals with walking and/or gait impairments in chronic stroke, there is still potential for further recovery with continued, intentional intervention.
Current treatment practice	
4	Key considerations when devising a treatment plan for walking and/or gait impairment for individuals with chronic stroke are ambulatory status, independence, insurance coverage, and caregiver availability.
Treatment limitations	
5	A heterogeneous population and lack of direct comparative evidence between treatment options cause barriers in developing standardized treatment guidelines for treating walking/gait impairment in chronic stroke.
7	There are critical barriers in treating walking/gait impairment in chronic stroke, including poor adherence, lack of accessibility (due to travel needs and availability of advanced treatment options), lack of insurance coverage, and concerns about safety.
Current treatments	
6	The most common treatments for walking/gait impairment in individuals with chronic stroke are overground gait training, treadmill training, balance training, and strength training, typically administered by a physical therapist.
16	Treatment options that may provide moderate/high clinical value for treating walking/gait impairment in chronic stroke, based on principles of motor relearning and exercise physiology, are general/combination physiotherapy, walking/exercise therapy, mobility-task training, and rhythmic auditory stimulation (RAS).
17	The following treatments have unclear/low clinical benefit for the treatment of walking/gait impairments in chronic stroke: acupuncture, virtual reality, over-the-counter supplements, antidepressant therapy, botulinum toxin, and functional electrical stimulation (FES).
18	Robot- or mechanical-assisted devices have low to minimal clinical benefit, may not be financially accessible for individuals, and can be bulky and difficult to use.
19	Ankle foot orthosis has moderate clinical benefit but have poor cosmesis and limited shoe choices.
20	Rhythmic auditory stimulation (RAS) is an effective therapy; however, access to this intervention is limited by the number of clinicians and/or socioeconomic factors.
Unmet need	
8	There is a need for improved education, so that individuals understand treatment goals, expected treatment progress, and the potential value of treatment, which may help to optimize engagement and participation.
9	Participation in treatment could be improved if individuals clearly understand how the treatment helps, see benefits associated with treatment, and find the treatment enjoyable.
10	An unmet need exists for treatments that can be delivered at home by a non-clinician to improve treatment accessibility and adherence, ensuring the regular practice required to obtain meaningful change in walking speed.
Valuable therapy	
11	The most critical features for a valuable treatment option for walking and/or gait impairment in chronic stroke include durability of treatment effect; safety; improvement in walking/gait speed, quality, endurance, and cosmesis; and treatment accessibility.
12	An optimal therapy for treating walking and/or gait impairments in chronic stroke includes sensors that monitor steps, walking speed, and/or distance to provide real-time feedback on treatment progress.
13	A potentially valuable therapy for treating walking and/or gait impairments in chronic stroke would be personalized to the individual, while being progressively challenging.
14	A potentially valuable therapy for treating walking and/or gait impairment in chronic stroke would provide periodic feedback about progress and support personal goal setting, which may help foster continued engagement in the treatment.
Future of therapy	
15	In an ideal world, individuals with walking and/or gait impairment in chronic stroke would have access to a multi-pronged, personalized treatment plan combined with an at-home, progressive treatment that provides feedback on progress.
Opportunity for improvement	
21	A treatment option employing RAS in a device that can be used at home, while monitoring treatment progress, providing feedback to the individual, and being continually progressive, could be valuable for individuals.

Appendix C

**Table 4:** Survey 3 Statements RAS, rhythmic auditory stimulation. *Agreement with statements was rated as “strongly disagree,” “disagree,” “neutral,” “agree,” or “strongly agree.”

Statement number	Survey 3 statements (polled statements are in bold)*
1	In the chronic phase of stroke, motor recovery is possible with appropriate interventions that challenge the individual at the appropriate level
2	In the chronic phase of stroke, a plateau in motor recovery may be an artifact resulting from discontinued interventions and/or the measurement tools currently available to clinicians
3	Effectively treating walking and/or gait impairments in individuals with chronic stroke should be a priority
4	Key considerations when devising a treatment plan for walking and/or gait impairments for individuals with chronic stroke include intervention efficacy, ambulatory status, patient independence, patient preference, insurance coverage, and caregiver availability
5	Treatment options that may provide moderate/high clinical value for treating walking/gait impairments in chronic stroke, based on principles of motor relearning, are general/combination physical therapy, therapeutic exercise, mobility-task training, and rhythmic auditory stimulation (RAS)
6	The US healthcare system structure imparts a critical barrier to following individuals with chronic stroke once discharged from formal acute rehabilitation, resulting from fragmented, uncoordinated care from multiple clinicians throughout the rehabilitation journey
7	An unmet need exists for therapeutic interventions that can be practiced safely outside of the clinic to improve treatment accessibility and participation
8	A valuable intervention for treating walking and/or gait impairments in chronic stroke would track gait quality metrics and walking speed and/or distance
9	Individuals with walking and/or gait impairment in chronic stroke should have access to a treatment plan that includes in-clinic and at-home practice with the goal of gradually increasing at-home practice while decreasing in-clinic practice as the individual progresses
10	The available evidence supports RAS as an effective therapy for improving walking and/or gait speed and quality (step cycle, step length, cadence, etc.) in individuals with chronic stroke
11	A treatment mechanism to deliver individualized RAS in a device that can be used safely at home could be valuable for individuals
12	There is an opportunity for education on and implementation of RAS amongst clinicians who treat individuals with chronic stroke

To view the RAS Scientific and Clinical Assessment Brief, please visit: https://shorturl.at/PgCCW.

## References

[1] Moore SA, Boyne P, Fulk G, Verheyden G, Fini NA (2022). Walk the talk: current evidence for walking recovery after stroke, future pathways and a mission for research and clinical practice. Stroke.

[2] de Rooij IJ, Riemens MM, Punt M, Meijer JG, Visser-Meily JM, van de Port IG (2021). To what extent is walking ability associated with participation in people after stroke?. J Stroke Cerebrovasc Dis.

[3] Khan F, Chevidikunnan MF (2021). Prevalence of balance impairment and factors associated with balance among patients with stroke. A cross sectional retrospective case control study. Healthcare (Basel).

[4] Donkor ES (2018). Stroke in the 21(st) century: a snapshot of the burden, epidemiology, and quality of life. Stroke Res Treat.

[5] Kim KT, Chang WK, Jung YS (2021). Unmet needs for rehabilitative management in common health-related problems negatively impact the quality of life of community-dwelling stroke survivors. Front Neurol.

[6] Price R, Choy NL (2019). Investigating the relationship of the functional gait assessment to spatiotemporal parameters of gait and quality of life in individuals with stroke. J Geriatr Phys Ther.

[7] Blennerhassett JM, Levy CE, Mackintosh A, Yong A, McGinley JL (2018). One-quarter of people leave inpatient stroke rehabilitation with physical capacity for community ambulation. J Stroke Cerebrovasc Dis.

[8] Bernhardt J, Hayward KS, Kwakkel G (2017). Agreed definitions and a shared vision for new standards in stroke recovery research: The Stroke Recovery and Rehabilitation Roundtable taskforce. Int J Stroke.

[9] Kennedy C, Bernhardt J, Churilov L (2021). Factors associated with time to independent walking recovery post-stroke. J Neurol Neurosurg Psychiatry.

[10] Khan F, Abusharha S, Alfuraidy A (2022). Prediction of factors affecting mobility in patients with stroke and finding the mediation effect of balance on mobility: a cross-sectional study. Int J Environ Res Public Health.

[11] Jorgensen HS, Nakayama H, Raaschou HO, Olsen TS (1995). Recovery of walking function in stroke patients: the Copenhagen Stroke Study. Arch Phys Med Rehabil.

[12] van de Port IG, Kwakkel G, van Wijk I, Lindeman E (2006). Susceptibility to deterioration of mobility long-term after stroke: a prospective cohort study. Stroke.

[13] Tilson JK, Settle SM, Sullivan KJ (2008). Application of evidence-based practice strategies: current trends in walking recovery interventions poststroke. Top Stroke Rehabil.

[14] Hornby TG, Reisman DS, Ward IG (2020). Clinical practice guideline to improve locomotor function following chronic stroke, incomplete spinal cord injury, and brain injury. J Neurol Phys Ther.

[15] Sall J, Eapen BC, Tran JE, Bowles AO, Bursaw A, Rodgers ME (2019). The management of stroke rehabilitation: a synopsis of the 2019 U.S. Department of Veterans Affairs and U.S. Department of Defense clinical practice guideline. Ann Intern Med.

[16] Stephan KM, Perennou D (2021). Mobility after stroke: relearning to walk. Clinical Pathways in Stroke Rehabilitation: Evidence-based Clinical Practice Recommendations.

[17] Teasell R, Salbach NM, Foley N (2020). Canadian Stroke Best Practice Recommendations: rehabilitation, recovery, and community participation following stroke. part one: rehabilitation and recovery following stroke; 6th edition update 2019. Int J Stroke.

[18] Jorgensen HS, Nakayama H, Raaschou HO, Vive-Larsen J, Stoier M, Olsen TS (1995). Outcome and time course of recovery in stroke. Part II: time course of recovery. The Copenhagen Stroke Study. Arch Phys Med Rehabil.

[19] Jorgensen HS, Nakayama H, Raaschou HO, Vive-Larsen J, Stoier M, Olsen TS (1995). Outcome and time course of recovery in stroke. Part I: outcome. The Copenhagen Stroke Study. Arch Phys Med Rehabil.

[20] (2014). Handbook of neurologic music therapy. Press.

[21] Ghai S, Ghai I (2019). Effects of (music-based) rhythmic auditory cueing training on gait and posture post-stroke: A systematic review & dose-response meta-analysis. Sci Rep.

[22] Damm L, Varoqui D, De Cock VC, Dalla Bella S, Bardy B (2020). Why do we move to the beat? A multi-scale approach, from physical principles to brain dynamics. Neurosci Biobehav Rev.

[23] Shang Z (2023). Use of Delphi in health sciences research: a narrative review. Medicine (Baltimore).

[24] Magee WL, Clark I, Tamplin J, Bradt J (2017). Music interventions for acquired brain injury. Cochrane Database Syst Rev.

[25] Lang CE, Bland MD, Connor LT (2011). The brain recovery core: building a system of organized stroke rehabilitation and outcomes assessment across the continuum of care. J Neurol Phys Ther.

[26] Dos Santos RB, Fiedler A, Badwal A (2023). Standardized tools for assessing balance and mobility in stroke clinical practice guidelines worldwide: a scoping review. Front Rehabil Sci.

[27] Winstein CJ, Stein J, Arena R (2016). Guidelines for adult stroke rehabilitation and recovery: a guideline for healthcare professionals from the American Heart Association/American. Stroke.

[28] (2021). Barriers and facilitators to engagement in rehabilitation among stroke survivors: an integrative review. Rehabil Nurs.

[29] Rice DB, McIntyre A, Mirkowski M, Janzen S, Viana R, Britt E, Teasell R (2017). Patient-centered goal setting in a hospital-based outpatient stroke rehabilitation center. PM R.

[30] Marín-Medina DS, Arenas-Vargas PA, Arias-Botero JC, Gómez-Vásquez M, Jaramillo-López MF, Gaspar-Toro JM (2024). New approaches to recovery after stroke. Neurol Sci.

